# Poultry Feather Waste as Bio-Based Cross-Linking Additive for Ethylene Propylene Diene Rubber

**DOI:** 10.3390/polym13223908

**Published:** 2021-11-12

**Authors:** Markus Brenner, Oliver Weichold

**Affiliations:** Institute of Building Materials Research, Schinkelstraße 3, 52072 Aachen, Germany; brenner@ibac.rwth-aachen.de

**Keywords:** keratin, composite, bio-based rubber additive, bio-based filler, cross-linking filler, waste reduction, green technologies, matrix-filler interaction

## Abstract

Most rubbers used today rely on sulphur as a cross-linking agent and carbon black from fossil resources to modify the mechanical properties. A very promising substitute can be found in natural keratins such as feathers. These are not only tough, but also contain a relevant amount of sulphur in the form of disulphide bridges. The present study shows that these can be activated under vulcanisation conditions and then bind covalently to EPDM rubber to form a cross-linked network. Feathers were cut into lengths of 0.08, 0.2, and 1 mm and incorporated at 38, 69, or 100 phr into EPDM mixtures containing either no carbon black or no carbon black nor sulphur. The presence of feather cuttings increases the tensile and compressive strength as well as the hardness, and reduces the rebound resilience. Due to their high (approximately 17%) nitrogen content, the feathers also improve the thermal stability of the composite, as the main degradation step is shifted from 400 °C to 470 °C and the decomposition is significantly slowed down. Since elastomers are a large market and feathers in particular are a high-volume waste, the combination of these two offers enormous ecological and economic prospects.

## 1. Introduction

One of today’s global challenges is the shift away from fossil resources and the general need to reduce waste generated by the economy [1]. Therefore, research into bio-based materials has intensified in recent years and several examples such as e.g., PLA or bio-PET are already available on the market [2]. These materials are often used in non-engineering applications such as food packing, but efforts to develop bio-based materials have also reached other industries e.g., the elastomer industry. One example is the development of a bio-based EPDM, which is produced in large quantities by e.g., Lanxess [3]. However, “bio-based” in this case refers only to the ethylene content, which comes from renewable resources and can contribute up to 75% of the EPDM terpolymer [4]. A large part of the final elastomer formulation consists of mainly petrochemical substances such as carbon black, process oil, and a complex mixture of cross-linking chemicals. Cross-linking of the EPDM terpolymer is generally accomplished by sulphur and sulphur-donating compounds such as dipentamethylenethiuram tetrasulphide [5], whereas the interaction of carbon black and the EPDM terpolymer mainly hinders the chain movement and, thereby, improves the material performance. On the whole, the petrochemical additives can make up more than half the elastomer weight [4]. Particularly the production of carbon black is an energy-intensive step with only low to moderate yields. This multiplies its environmental impact. Globally more than 8 million tons of carbon black are produced every year, most of which are used as filler for the rubber and polymer industries [6]. As a consequence, some research efforts focused on methods to produce carbon black from bio-based feedstocks [7] or to evaluate the potential of other bio-based alternatives such as walnut shells [8] or fish scales [9]. Yet, none of these have gained access to the market on a larger scale. Another potential source for renewable bio-based fillers are poultry feathers. Approx. 4 million tons are produced every year and increasing meat consumption will most likely lead to a further increase in the future [10]. These amounts accumulate as expensive biogenic waste that are disposed of in landfills.

Despite their reputation as waste, feathers are a fascinating material. They consist almost entirely of the scleroprotein keratin. The exceptional characteristic of keratin is the cross-linked structure provided by intra- and intermolecular disulphide bridges between the cysteine units of the protein chains. Keratins, therefore, have a high sulphur content [11]. In hydrolysed keratins, this sulphur content is available for chemical reactions. Yue et al. showed that derivatives of norbornene, which is sometimes used as diene in EPDM terpolymers, can be cross-linked by hydrolysed hair keratin [12]. Since both EPDM and keratin gain their unique material properties from being cross-linked by sulphur bridges, the question arises as to whether keratin can be used to cross-link EPDM rubbers. Since poultry feathers are considered as waste, their usage is almost CO_2_ neutral and, taking the costly harmful landfill into account, could even lead to a negative CO_2_-footprint. Since keratin is fibrous, it could also possibly act as covalently-bound reinforcement and thereby improve the mechanical properties of the elastomer. Moreover, keratin is expected to improve the fire behaviour such as flammability, combustibility, and burning rate. Similar effects of keratin were recently shown for keratin-impregnated wood fibre boards [13] and for keratin-fibre containing thermosets [14].

To test the feasibility of feathers as a filler for EPDM rubber, a standard, industry-proven EPDM recipe was used. In the following experiments, carbon black was completely substituted by goose feather cuttings in three different lengths (0.08, 0.2 and 1 mm). To the best of our knowledge, studies investigating the interaction of feathers with EPDM are not known in literature. Therefore, this research investigates whether feathers can be used as a cheap bio-based filler in EPDM and to what extent feathers can improve the materials’ performance due to their unique hierarchical structure and the unique disulphide cross-linking chemistry of keratin.

## 2. Materials and Methods

Goose feathers were obtained from a feather and down company (Seibersdorfer Bettfedern- und Daunenfabrik GmbH, Wimpassing an der Leitha, Austria) where they accumulate in chamber 1 during the process of bead feather and down sorting. These feathers have a length of between 45 and 90 mm and a shaft diameter of 0.6 to 1.45 mm (see Figure S1 in the Supporting Information). The material was cut to lengths of 1 mm and 0.2 mm using a SM200 cutting mill (Retsch GmbH, Haan, Germany) equipped with the corresponding sieves (see Figure S2 in the Supporting Information). The 0.08 mm fraction was produced on a Pulverisette 14 premium line (Fritsch GmbH, Idar-Oberstein, Germany) in cutting mode with a 0.08 mm sieve. Three elastomer mixtures were provided by CARLISLE^®^ Construction Materials GmbH (AD, Kampen, The Netherlands). The original mixture contained 34.08% ethylene propylene diene termonomer (EPDM), 34.08% carbon black, 29.31% paraffin oil, 1% zinc oxide (ZnO), 0.41% stearic acid, 0.41% sulphur, 0.41% *N*-cyclohexyl-2-benzothiazolesulfenamide (CBS) and 0.27% *N*,*N*-dicyclohexyl-2-benzothiazolsulfene amide (TBzTD). Derived from this, a mixture without carbon black (M1) and a second one containing no carbon black or sulphur (M2) were used (Table 1). Toluene (>99%) was purchased from VWR GmbH and Co. KG (Kampen, Germany).

### 2.1. Preparation of Feather Elastomer Mixtures and Vulcanisation

The cut feathers were mixed with the elastomer at 80 °C using a Haake Rheomix Type RC 300P laboratory kneader (Thermo Scientific, Waltham, MA, USA) equipped with R600 Banbury rotors using a rotation speed of 20 rpm. In all experiments, the mixing chamber was only filled to 85 vol% of the maximum volume (78 cm^3^) to guarantee reproducible mixing conditions. In a typical experiment, the appropriate EPDM-mixture was fed into the kneader. The feathers were added as soon as the mixture reached 80 °C. Kneading was continued for 5 min, before being removed from the kneader. This mixture was rolled into 2 mm sheets using a laboratory rolling mill with two rollers (COLLIN Lab & Pilot Solutions GmbH, Maitenbeth, Germany). These sheets were cut into 50 × 80 mm^2^ rectangles, which were then used to prepare 2 mm thick, vulcanized samples for tensile tests, rheological measurements, and Soxhlet extractions. Alternatively, the rolled sheets were cut into 50 × 50 mm^2^ rectangles. Three of these were stacked and compressed to prepare 5 mm-thick samples for hardness, rebound, and compression tests. Vulcanisation was accomplished in a 300P hot press (COLLIN Lab & Pilot Solutions GmbH, Maitenbeth, Germany) at 150 °C for 30 min using appropriately sized steel moulds (50 × 80 × 2 mm^3^ and 50 × 50 × 5 mm^3^) and 184 kN closing force (machine pressure 150 bar). After vulcanisation, the samples were stored for at least 24 h at room temperature before further use.

### 2.2. Hardness and Rebound Resilience

Shore-A hardness and rebound resilience were tested on 5 mm-thick, vulcanized samples. Shore hardness was measured following DIN 53505 using a Shore-A hardness tester from Bareiss Prüfgerätebau GmbH, Oberdischingen, Germany. For this, the tester was pressed onto the elastomer sample and the value displayed after 3 s was noted. Measurements were repeated in nine different spots for each material.

The rebound resilience was tested following DIN EN ISO 8307. For this, a metal ball with a weight of 16.9 g was dropped onto the sample from a height of 50 cm in a pipe with an inner diameter of 30 mm. The height of the first rebound was noted and the measurement was repeated 9 times at different spots on the sample. If the ball touched the pipe on its way down or up, the measurement was discarded.

### 2.3. Uniaxial Tensile Test

Tensile tests were carried out on an Instron 5566 universal testing machine following DIN 53504 using dog-bone shaped samples according to DIN EN ISO 527-2 type 1A. These were punched out of the 2 mm thick vulcanised sheets. For each mixture three samples were made and tested. The tests were run strain-controlled using a strain rate of 200 mm/min. Recording started when the applied load reached 2 N.

### 2.4. Compression Test

Compression tests were performed using the Allround-Line table top test machines Z150 and Z100 (ZwickRoell GmbH & Co. KG, Ulm, Germany). For each mixture, three samples with a dimension of 50 × 50 × 5 mm^3^ were tested. Prior to testing, the samples were powdered with talcum on both sides. The sample was placed between two metal plates and the measurement started when the applied load reached 10 N. Samples were compressed by 40% of the original height within 1 min and afterwards relaxed to the starting force (10 N) at the same rate. The compression was repeated three times during which the applied force and displacement of the cross beam were recorded.

### 2.5. Small Amplitude Oscillatory Shear (SAOS) Experiments

Small amplitude oscillatory shear (SAOS) measurements were conducted using a MCR 102 rheometer (Anton Paar GmbH, Graz, Austria). Circular samples with a diameter of 25 mm were punched out of the 2 mm thick plates. The storage modulus was measured in an isothermal experiment at 20 °C with an angular frequency of 1 Hz and an amplitude of 0.02%. In every experiment 100 points were recorded.

### 2.6. Attenuated Total Reflection–Fourier Transform Infrared Spectroscopy (ATR-FTIR) Measurements

Infrared spectra were recorded using a Spectrum Two UATR FTIR spectrometer (Perkin Elmer Inc., Waltham, MA, USA) equipped with a diamond ATR (attenuated total reflection) window. All spectra were recorded in the spectral range of 400–4000 cm^−1^ with 12 scans at a spectral resolution of 4 cm^−1^. Before each measurement, the diamond ATR crystal was cleaned with isopropanol.

### 2.7. Thermogravimetric Analysis (TGA)

TGA measurements were performed on a TGA 4000 (Perkin Elmer Inc., Waltham, MA, USA) instrument heating from 30 °C to 850 °C at a rate of 10 K/min under a continuous flow of oxygen at a flow rate of 20 mL/min. Samples were stored at 21 °C and 55% RH prior to the measurements.

### 2.8. Soxhlet Extraction of Vulcanised EPDM without Sulfur

The sulphur-free EPDM mixture (M2) was mixed with 100 phr feathers and vulcanised into a 2 mm thick sheet as described above. The sheet was cut into strips and approximately 3 g of these strips were put into a Soxhlet extractor filled with 200 mL of toluene. The oil bath was heated to 145 °C to produce a constant toluene backflow within the Soxhlet extractor resulting in at least two turnovers per hour. Extraction was carried out for 72 h. Afterwards, samples were dried and weighed to calculate the residual mass. For this, the feathers were considered as not extractable, while paraffin oil was considered as completely extracted. Accelerator, activator, and other substances were not included in the calculation as they account for less than 2% of the mixture and they undergo several potential reactions in the vulcanisation process. The mass of residual EPDM $m_{R}$ was, therefore, calculated following Equation (1):

(1) $$m_{R}=100\cdot \frac{m_{\mathrm{residue}}-m_{\mathrm{feathers}}}{m_{\mathrm{EPDM}}}$$

### 2.9. Scanning Electron Microscopy (SEM)

Prior to SEM measurements samples were coated with a 10 nm thick layer of gold-palladium using a sputter coater (Leica EM SCD500, Leica, Wetzlar, Germany). SEM experiments were performed with an ESEM XL30 FEG (FEI, Phillips, Amsterdam, The Netherlands) with a voltage of 2.7 kV. Freeze-fractured samples were cooled with liquid nitrogen and broken by using a steel hammer.

## 3. Results and Discussion

Feathers were cut into 0.08, 0.2, and 1 mm lengths and individually mixed with the crude rubber mixture containing no carbon black (M1). Before adding the feathers, this rubber mixture exhibited poor workability and stuck to the rollers of the rolling mill rather than forming a uniform sheet. This was not the case after the addition, but the feathers were still visible to the naked eye, especially at higher content. The maximum amount of feathers used was 100 phr, which is equivalent to the amount of carbon black in the original mixture. Higher contents are possible, but quickly reduce the workability. After vulcanisation at 150 °C for 30 min, the feathers were barely noticeable. Qualitatively, the obtained materials appeared rather stiff.

**Figure 1:**
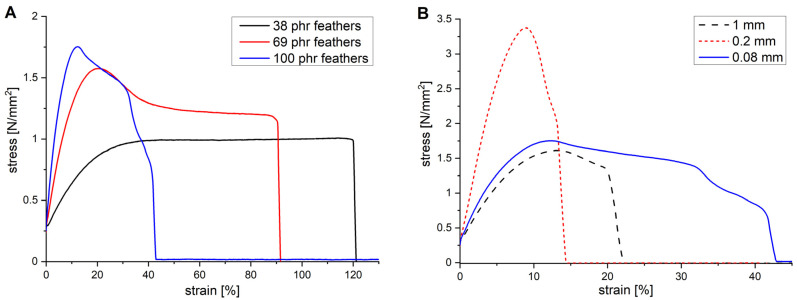
Stress–strain curves of the vulcanised EPDM mixture M1 filled with increasing amounts of 0.08 mm feathers cuttings (**A**) and with 100 phr feather cuttings of different length (**B**). Specimens were prepared from a single, 2 mm-thick sheet.

Examples of the stress–strain curves from tensile tests of the vulcanised mixtures containing feather cuttings are shown in Figure 1. The full set is presented in Figure S3 in the Supporting Information. At low feather contents, the curves are governed by large deformations without pronounced yield points (Figure 1A). These are only observed at higher contents, in our set at 69 and 100 phr. With increasing feather content, the yield point shifts to lower strains and higher stresses, indicating increased stiffness and strength. This is a first indication that external loads can be transferred to the feathers and these can act as reinforcement. When comparing the three lengths at e.g., 100 phr (Figure 1B), the 0.2 mm cuttings show the best performance in terms of stiffness and strength, while the 0.08 mm cuttings give rise to a larger area of plastic deformation than the 1 mm cuttings. The latter in particular is unexpected as longer fibres have a greater embedded length and usually exhibit a more pronounced fibre pull-out. Considering the situation at the fibre/matrix interface, the tensile stress σ inside the sample leads to a shear stress τ at the interface. The load *P* required to pull a fibre out of the matrix is *P* = 2 π *r* *l* τ, with *r* and *l* being the fibre radius and the embedded length. The higher strength and stiffness of the samples containing 0.2 mm cuttings compared to those containing 0.08 mm cuttings can, therefore, be explained by the greater embedded length. However, the pull-out load is balanced by the tensile strength σ_f_ of the fibre according to:

σ_f_ π *r*^2^ = 2 π *r*
*l τ.* (2)

The length at which *P* equals the tensile strength of the fibre is called the critical length *l*_c_ and at *l* > *l*_c_, the fibre breaks. Assuming that for the 0.2 mm fibres *l* < *l*_c_, the increase in tensile strength of the vulcanised composite can also be interpreted in such a way that, due to the greater embedded length, more of the tensile strength of the feathers is activated. Rearranging Equation (2) affords:

(3) $$\frac{l}{r}=\frac{\sigma_{f}}{2\tau }.$$

That is, the performance of fibres as reinforcement is a function of their aspect ratio. In composites using short synthetic fibres as reinforcement, this ratio is usually systematically increased by increasing *l*. However, feathers are hierarchically structured and contain, besides the massive quill, also very delicate barbules and barbicels in the vanes. Therefore, it is possible that from the 0.2 mm to the 1 mm fraction, the aspect ratio actually becomes smaller due to a disproportionate increase in *r* as thicker parts of the calamus may pass the 1 mm exit sieve of the mill in the cutting process. This does not seem possible in the case of the 0.2 mm sieves, where the area of the individual sieve opening is approximately 25 times smaller than that of the 1 mm sieve. In contrast, the factor from the 0.2 mm to the 0.08 mm opening is only approximately 6.25 and so the aspect ratio of these two fractions is expected to be similar and, hence, the tensile behaviour follows the expected trend.

**Figure 2:**
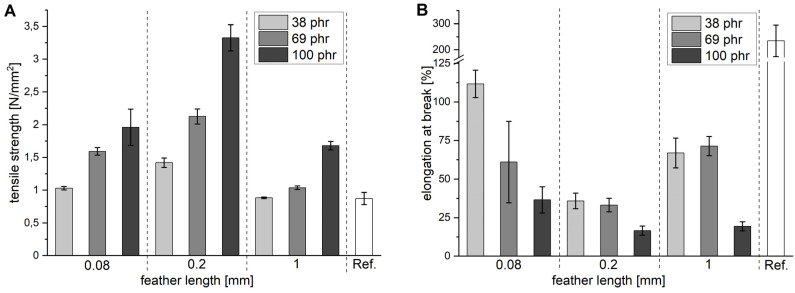
Tensile strength (**A**) and elongation at break (**B**) of the vulcanised EPDM mixture M1 as a function of the amount and length of the feather cuttings. Specimens were prepared from a single, 2 mm thick sheet. A vulcanised M1 mixture without feathers is used as reference. Note the interruption of the y-axis at the reference in (**B**).

On the whole, the tensile strength increases with increasing amount of feather cuttings for all lengths (Figure 2A). The effect is most pronounced for the 0.2 mm fraction, which at a content of 100 phr exceeds the reference by a factor of approximately 3.5. At the same time, the elongation at break point decreases with increasing fibre length, and from the 0.08 to the 0.2 mm fraction (Figure 2B). The results in Figure 2 are consistent with the above explanations and show even more clearly than Figure 1 that the 1 mm fraction does not fit into the overall trend.

**Figure 3:**
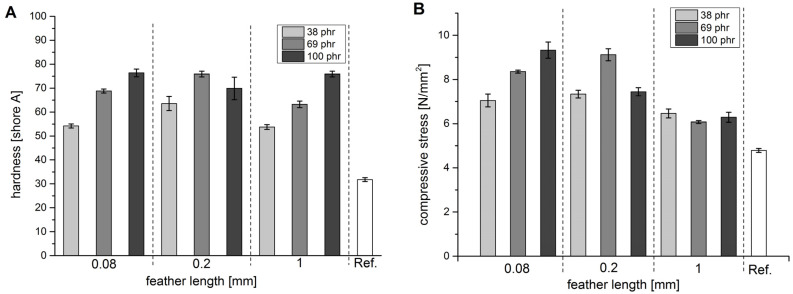
Hardness of the vulcanised M1 mixture with increasing amount of feather cuttings (**A**); compressive stress measured under uniaxial compression to 40% of the original height of the sample (**B**). A vulcanised M1 mixture without feathers is used as reference. Specimens were prepared from 5 mm thick samples.

The shore hardness increases with increasing feather content for all lengths (Figure 3A). The only exception is the mixture containing 100 phr of the 0.2 mm feathers. In terms of hardness, 0.2 mm seems to be the best length, but this particular mixture suffers from poor workability. Apparently, 100 phr of 0.2 mm cuttings could not be distributed homogeneously in the rubber mixture (see Figure S4 in the Supporting Information). This is quite curious as 100 phr of the 0.08 mm cuttings, which have a larger total surface area, could be distributed evenly. In addition, the stack of 2 mm sheets did not consolidate defect-free into a homogeneous 5 mm plate during vulcanisation (see Materials and Methods for a description). Rather, the individual layers remained separated in a number of places, which resulted in a lower apparent hardness. Better results could, potentially, be obtained by adding more paraffin oil to assist in dispersing the fibres. As a consequence, the highest values in shore hardness were obtained with 100 phr 0.08 mm and 69 phr 0.2 mm cuttings. Both values were between 75 and 80, which was more than twice the value of the reference without feathers (31, empty bar on the right) and almost 40% higher than mixtures with 100 phr carbon black (56, not shown).

The compression behaviour was assessed by comparing the stress at a compression of 40% of the original height (Figure 3B). For the 0.08 and 0.2 mm cuttings, the observed trend was similar to that found for the hardness (Figure 3A). The values increased steadily with increasing amounts of feathers, with the sample containing 100 phr of 0.2 mm cuttings again being the exception for the reasons outlined above. Compared to the reference without feathers, the force almost doubled for the best samples (again 100 phr 0.08 mm and 69 phr 0.2 mm cuttings) and exceeded the sample with 100 phr carbon black by 20% (not shown). The samples containing 1 mm cuttings showed a different behaviour. Adding 1 mm feather cuttings increased the compressive strength by about 35% compared to the reference, but this appeared to be virtually independent of the amount. Since this length was found to be a poor tensile reinforcement (Figure 2A), the main contribution appeared to originate in the compressive strength of the feathers, similar to the mode of action of aggregates in concrete.

**Figure 4:**
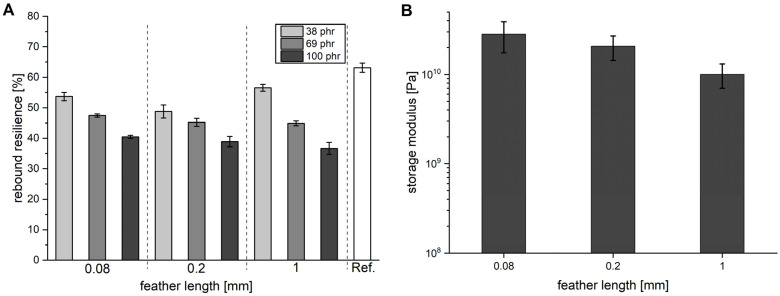
Rebound elasticity of the vulcanised M1 mixture with increasing amount of feather cuttings (**A**). A vulcanised M1 mixture without feathers is used as reference; Storage modulus of vulcanised M1 mixtures containing 100 phr feather cuttings measured by small amplitude oscillatory shear (SAOS) experiments at 20 °C (**B**). Specimens were prepared by stacking three 2 mm thick sheets prior to vulcanisation.

To further understand how feathers influence the elastic properties of the material, the rebound resilience was measured following DIN EN ISO 8307 (Figure 4A). The value indicates how much kinetic energy is returned by the rubber after an impact. The highest rebound resilience (63%) was observed for the reference without feathers. Increasing the amounts of feathers continuously decreased the rebound resilience without much difference between the three lengths and was lowest at 100 phr. Generally, increasing the filler content resulted in an increased hardness and a reduced rebound resilience. Similar effects were observed when adding carbon black, for which a value of 49 was found at 100 phr. As rubbers are entropy elastic materials, both observations can be explained by the fact that fillers hinder the chain movement and increase the internal friction. With increasing filler content, more energy is dissipated in the material and less is returned. In addition, samples with more filler contain less of the elastomer, which also has a negative effect on the elastic properties. Contrary to, for example, the tensile strength, the rebound resilience at 100 phr appeared to decrease in the order 0.08 mm > 0.2 mm > 1 mm. This observation was corroborated by SAOS experiments (Figure 2B), which indicated that the storage modulus decreased in that order. As SAOS experiments only induce small deformations, fibre fracture was not expected, so this appeared not to be a question of the aspect ratio. The increased storage modulus was, therefore, most likely an effect of the larger surface area attributed to the smaller feather cuttings. This increased the interaction with the rubber matrix and simulated a higher crosslinking density. The resulting denser network can store more energy, while the energy dissipation through internal friction is unchanged. The effect was not as prominent as would be expected for bulk materials, because feathers already have a highly hierarchical structure as already stated above in the description of the aspect ratio.

**Figure 5:**
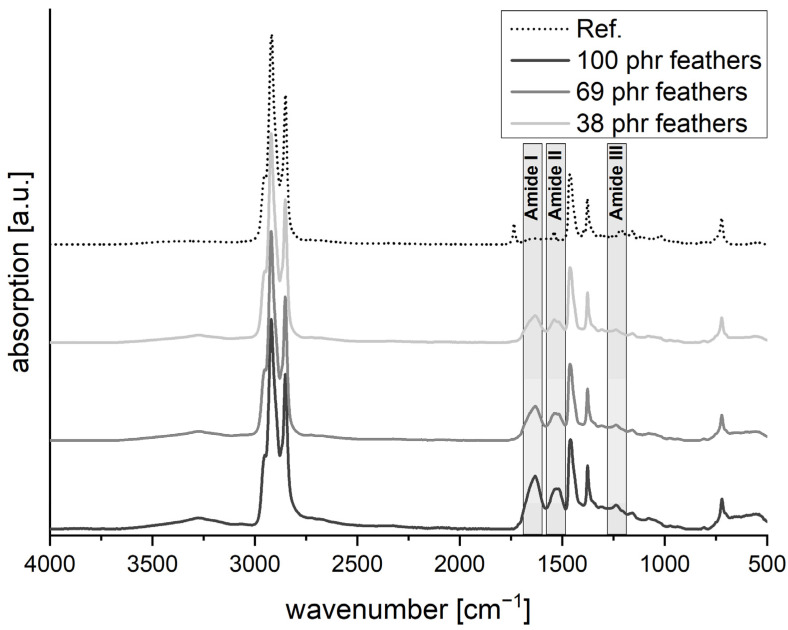
Attenuated total reflection–Fourier transform infrared spectroscopy (ATR-FTIR) analysis of vulcanised EPDM (M1) filled with increasing amounts of 0.08 mm feathers. A vulcanised M1 mixture without feathers is used as reference.

Samples containing 0.08 mm feathers were used for IR analysis as these should have the highest homogeneity (Figure 5). The reference without feathers shows the typical spectrum for vulcanised EPDM with C–H stretching vibrations in the region between 2800 and 3000 cm^−1^ and C–H bending between 1350 and 1450 cm^−1^. No major bands are seen in the C=C double bond regions from 3000 to 3200 cm^−1^ or 1500 to 1700 cm^−1^, which would indicate the presence of unvulcanised material. With the addition of feathers, the amide I, II and III bands of the protein at 1650, 1550 and 1230 cm^−1^ become increasingly pronounced as the feather content increases. This is a sign of homogeneous distribution of the feathers within the rubber matrix. In addition, the bands at approximately 3250 cm^−1^ also slightly increase with an increasing amount of feathers. This is the typical region for O−H and N−H stretching vibrations, so that these bands could come from the peptide bonds, protein-bound serine or tyrosine, or moisture.

**Figure 6:**
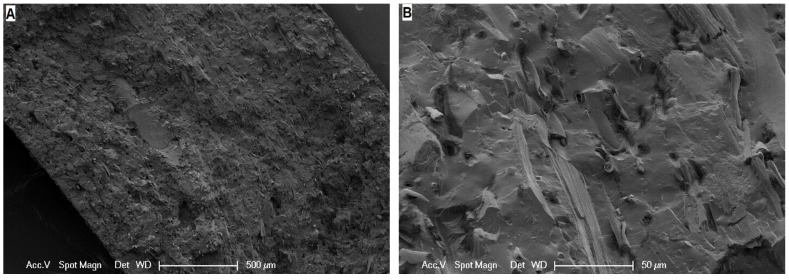
Scanning electron microscopy (SEM) images of a freeze fractured sample of M1 containing 100 phr of 0.08 mm feathers with 50× (**A**) and 500× (**B**) magnification.

To validate the homogenous distribution of the feather cuttings, the samples were freeze-fractured and analysed using scanning electron microscopy (Figure 6). At 50× magnification, a homogenous distribution of feathers in the sample is visible and no agglomerates can be seen. The 500× magnification reveals that most of the fine feather fibres are torn in the fracture plane, which indicates a good matrix fibre adhesion. Still, some of the fibres are pulled out of the elastomer matrix as seen from the empty holes or fibres sticking out.

**Figure 7:**
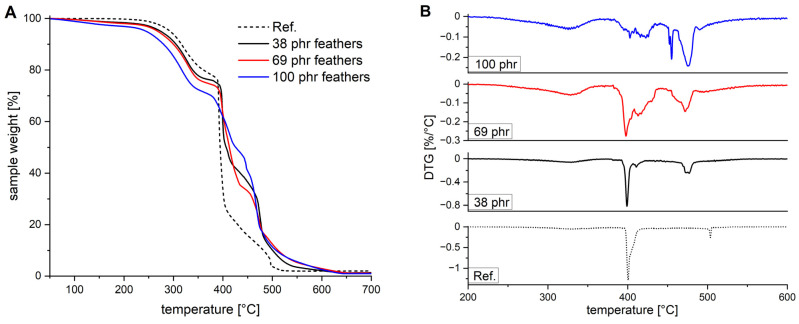
Thermogravimetric analysis (TGA) data (**A**) and DTG (**B**) of EPDM (M1) filled with different amounts of 0.08 mm feathers heated with 10 °C /min under oxygen atmosphere. A vulcanised M1 mixture without feathers is used as reference.

Thermogravimetric analyses in an enriched oxygen atmosphere were used to investigate the interaction of feathers and EPDM in terms of thermal degradation (Figure 7). In the region up to 200 °C, the weight loss increases with increasing amounts of feathers. This is most likely attributed to evaporating water—the equilibrium humidity in feathers is up to 8 wt%—which explains the nature of the increasing bands at approximately 3250 cm^−1^ in the IR analyses (Figure 5). The increasing amount of moisture could potentially be favourable as it should facilitate biodegradation. Furthermore, the presence of moisture could reduce the tendency to swell in non-polar solvents, one of the mayor drawbacks of EPDM rubbers. Between 300 and 385 °C, a slow degradation step is observed for all mixtures associated with a weight loss of approx. 20%. This corresponds to the amount of paraffin oil in the mixture. At about 400 °C, the reference shows rapid degradation losing another 60% of its weight within the next 20 °C. With the addition of feathers, this rapid degradation increasingly slows down. With 100 phr of feathers, the main degradation is shifted to 470 °C and proceeds at a much slower rate. Total degradation of the reference is reached at about 500 °C, while for the mixture with 100 phr feathers, the degradation ends at about 650 °C. The change in the degradation behaviour above 400 °C from instantaneous combustion to gradual decomposition indicates the positive effect of the protein, which most likely originates in a chemical interaction.

**Figure 8:**
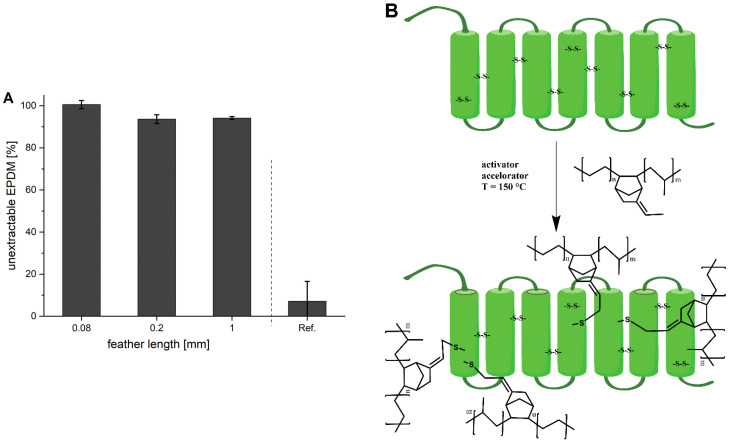
(**A**) Amount of non-extractable material after 72 h of Soxhlet extraction with toluene. Samples were prepared with 100 phr of feathers in three lengths using the EPDM mixture M2 without sulphur. A vulcanised M2 mixture without feathers and sulphur is used as reference. (**B**) Schematic illustration of the cross-linking of EPDM rubber with keratin.

A number of the aforementioned results indicate a chemical interaction of feathers and EPDM. As stated above, feather keratin contains 1.5% of sulphur in the form of cysteine disulphide bridges [15] and EPDM is usually cross-linked using sulphur. The natural assumption is, therefore, that EPDM reacts with keratin at the disulphide bridges. To test this assumption, samples were prepared using the EPDM mixture M2, which does not contain any sulphur and, thus, cannot cross-link by itself under vulcanisation conditions. The test is then based on the fact that unvulcanised EPDM dissolves in toluene, while vulcanised EPDM rubber does not. Figure 8A clearly shows that mixtures with 100 phr of feather cuttings contain almost no extractable EPDM, while the reference without feathers is almost completely dissolved. This indicates the enormous extent to which feathers can act as cross-linker in this system. Upon close examination, the amount of non-extractable material appears to increase slightly as the length decreases from 0.2 to 0.08 mm. This might again be due to the larger surface area and, with it, the increased feather–matrix interaction.

**Figure 9:**
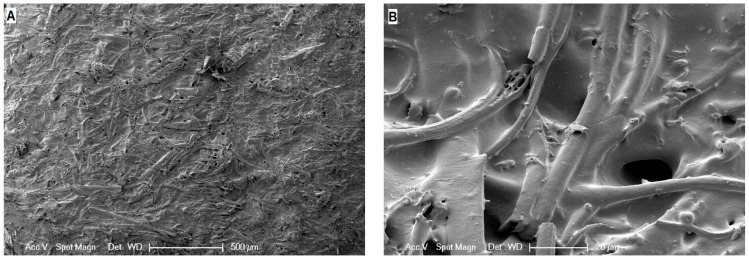
SEM images of vulcanized samples from EPDM mixture M2 without sulfur after extraction with toluene for 72 h. 50× magnification of the mixture with 68 phr of 80 µm feathers on the (**A**) and 1000× magnification of the mixture with 100 phr of 200 µm feathers on the (**B**).

Although the extraction test is not definitive proof of the formation of sulphur bridges between EPDM and the feathers, this is highly likely and in agreement with previous reports e.g., by Yue et al. [12]. A further indication for covalent bonds between feathers and the rubber is provided by SEM analysis of the extracted samples (Figure 9). Although the feathers are clearly visible, they are still fully covered by the rubber and gaps between the feathers are filled with rubber. It should be mentioned again that the samples shown in Figure 9 were extracted with toluene for 72 h and the rubber mixture did not contain any sulphur for cross-linking. This is very strong support for our hypothesis of covalent bonds being formed between EPDM and feathers.

Figure 8B shows an illustration of the possible reaction. The disulphide bridges in keratin are cleaved and both fragments add to the allylic position of the EPDM chain [16]. Based on the substitution pattern, the reaction appears to proceed by a radical mechanism, similar to e.g., the Wohl-Ziegler bromination. However, disulphide bonds are known to differ in their accessibility and reactivity depending on the chemical environment [17,18]. Therefore, it is possible that not all of the disulphide bridges are able to react with EPDM.

## 4. Conclusions

Both poultry feathers and vulcanised EPDM rubber gain their unique material properties from being cross-linked by sulphur bridges. The results shown here indicate that conventional vulcanisation chemicals can activate the disulphide bridges in protein materials to cross-link unsaturated rubbers and that hydrolysis of the protein is not necessary. The incorporation of intact feather cuttings, thus, makes use of all material properties associated with the feathers, not only the chemical reactivity. As a result, a substantial improvement of the mechanical and thermal properties was found for the composites. Feathers affect the EPDM rubber far more as an inactive filler would. Due to these unique properties, keratinous materials have the potential of becoming a new class of fillers for sulphur vulcanised rubbers with the intrinsic ability to actively crosslink with the rubber. Using structures already built and optimised by nature, in this case feathers with intact hierarchical organisation of high molecular weight proteins, is far more resource-efficient and, thus, more economical and ecological than going through the effort of assembling complex structures from small molecules even if these come from bio-based feedstock.

## Figures and Tables

**Table 1:** Compositions of the original EPDM mixture and the two mixtures M1 and M2 derived from it.

Components	Original Mixture		M1	M2
	Amount/phr	Amount/wt%	Amount/phr	Amount/phr
EPDM	100.0	34.08	100.0	100.0
Carbon Black	100.0	34.08	0	0
Paraffin Oil	86.0	29.31	86.0	86.0
ZnO	3.0	1.02	3.0	3.0
Stearic acid	1.2	0.41	1.2	1.2
Sulphur	1.2	0.41	1.2	0
CBS 80%	1.2	0.41	1.2	1.2
TBzTD 80%	0.8	0.27	0.8	0.8
Total	293.4	100.00	193.4	192.2

## Data Availability

The data presented in this study are available on request from the corresponding author.

## Supplementary Materials

The following are available online at https://www.mdpi.com/article/10.3390/polym13223908/s1, Figure S1: Goose feathers as received before cutting, Figure S2: The three lengths of shredded feathers cuttings used in the study, Figure S3: Exemplary stress–strain curves for EPDM mixtures with different amounts of 0.2 mm feathers (A) and 1 mm feathers (B), Figure S4: Cross-section of the 5 mm sample with 100 phr of 0.2 mm feather cuttings showing incomplete consolidation during vulcanisation.
