# Supplementary material for: Poultry Feather Waste as Bio-Based Cross-Linking Additive for Ethylene Propylene Diene Rubber
Source: Polymers (Basel). 2021 Nov 12;13(22):3908. doi: 10.3390/polym13223908 (PMC8623415; doi:10.3390/polym13223908)
Supplement: Supplementary file 1 [file polymers-13-03908-s001.zip › polymers-1436476-supplementary.pdf]

Supplementary Material

to

Poultry feather waste as bio-based cross-linking additive for EPDM rubber

Markus Brenner and Oliver Weichold

Institute of Building Materials Research, Schinkelstraße 3,
52072 Aachen, Germany

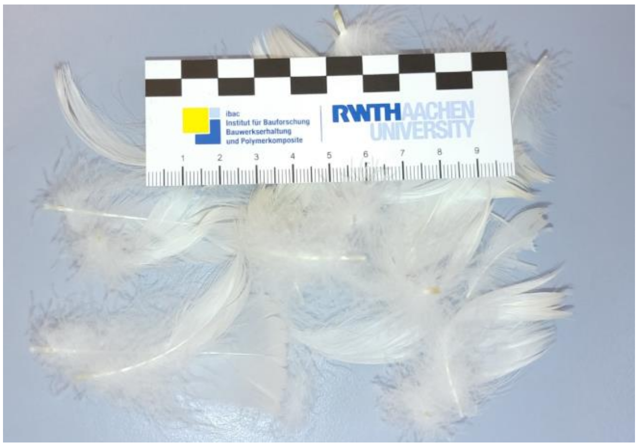

Figure S1. Goose feathers as received before cutting.

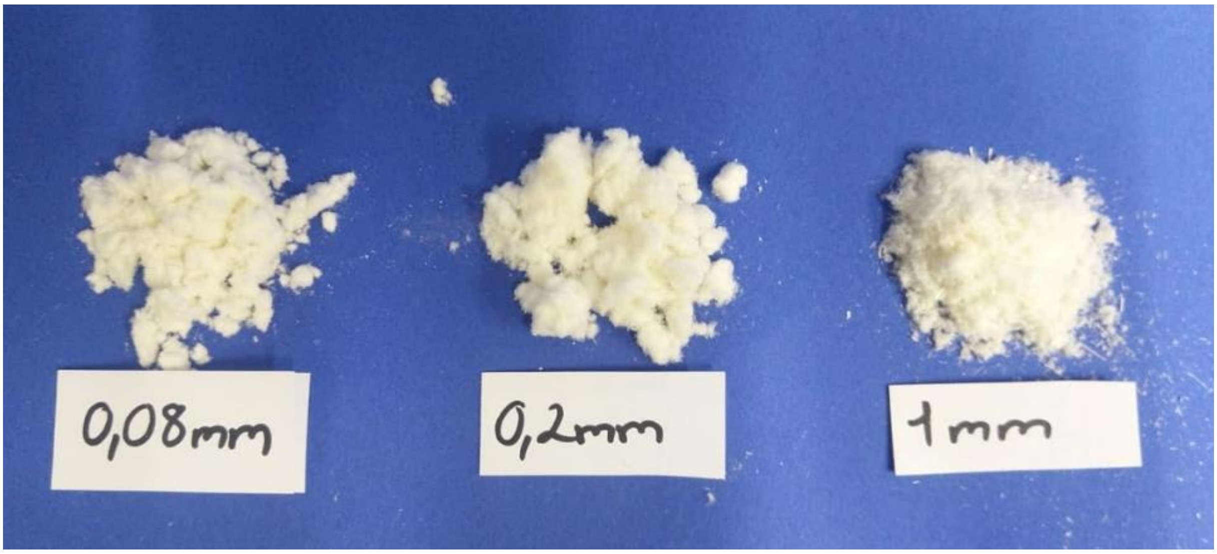

Figure S2. The three lengths of shredded feathers cuttings used in the study.

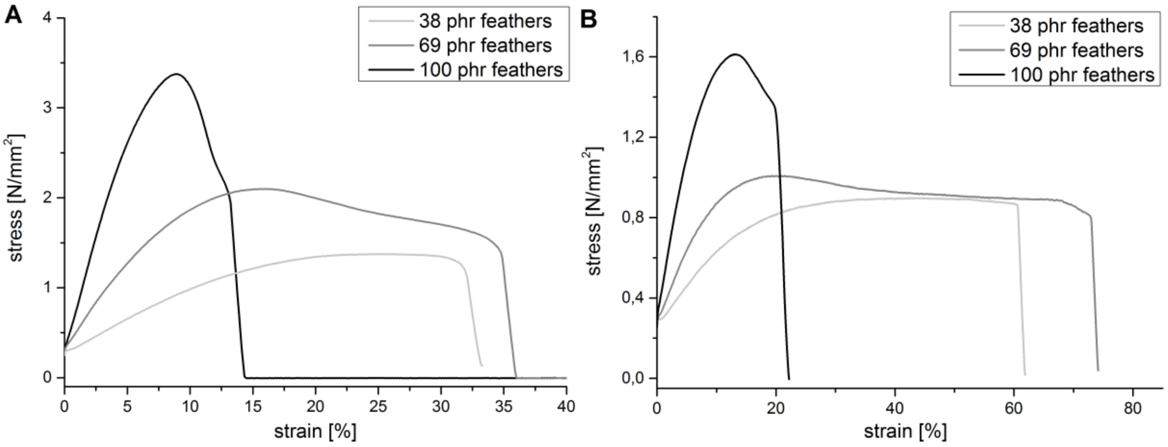

Figure S3. Exemplary stress-strain curves for EPDM mixtures with different amounts of 0.2 mm feathers (A) and 1 mm feathers (B).

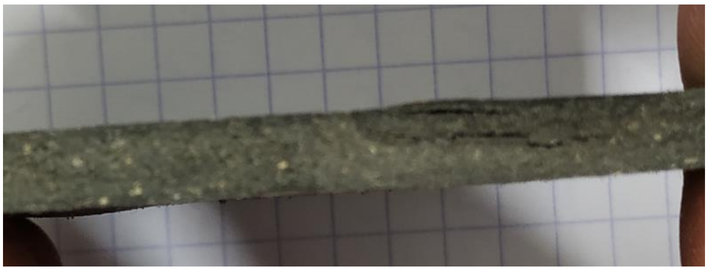

Figure S4. Cross-section of the 5mm sample with 100 phr of 0.2 mm feather cuttings showing incomplete consolidation during vulcanisation.
